# Contextual Barriers and Facilitators to Implementing Ultrasound-Guided Fine-Needle Aspiration and Rapid Onsite Evaluation in a Resource-Limited Setting

**DOI:** 10.1200/GO-25-00718

**Published:** 2026-06-10

**Authors:** Asteria H. Kimambo, Edda Vuhahula, Elia J. Mmbaga, Balowa Musa Baraka, Atuganile Malango, Zainab Illonga, Beatrice P. Mushi, Bhoke Kitosho, Katherine Van Loon, Dianna L. Ng

**Affiliations:** ^1^ Department of Pathology, Muhimbili University of Health and Allied Sciences, Dar es Salaam, Tanzania; ^2^ Hubert Kairuki Memorial University, Dar es Salaam, Tanzania; ^3^ Department of Epidemiology and Biostatistics, Muhimbili University of Health and Allied Sciences, Dar es Salaam, Tanzania; ^4^ Department of Community Medicine and Global Health, University of Oslo, Oslo, Norway; ^5^ Department of Radiology and Imaging, Muhimbili University of Health and Allied Sciences, Dar es Salaam, Tanzania; ^6^ Department of Pathology, Muhimbili National Hospital, Dar es Salaam, Tanzania; ^7^ Helen Diller Family Comprehensive Cancer Center, University of California, San Francisco, San Francisco, CA; ^8^ Department of Medicine, Division of Hematology/Oncology, University of California, San Francisco, San Francisco, CA; ^9^ Department of Pathology and Laboratory Medicine, Memorial Sloan Kettering Cancer Center, New York, NY

## Abstract

**PURPOSE:**

Fine-needle aspiration (FNA) is an inexpensive, minimally invasive tissue sampling method that is often the primary diagnostic procedure in low- and middle-income countries. Although FNA can yield highly accurate diagnostic samples when performed by skilled practitioners, inadequate training frequently leads to indeterminate, nondiagnostic, or false-negative results. The adequacy and accuracy of FNA can be significantly improved by using image guidance and rapid onsite evaluation (ROSE). This study assessed the barriers to and facilitators of ultrasound-guided FNA (USFNA) and ROSE at a national referral hospital in Tanzania.

**METHODS:**

We conducted situational analysis using semistructured interviews with multiple key stakeholders involved in cancer care, including pathologists, surgeons, interventional radiologists, laboratory scientists, and administrators. The prompts included questions about barriers and facilitators for implementing the USFNA and ROSE. The interviews were recorded, transcribed, translated, and coded using thematic context analysis, and major themes were identified.

**RESULTS:**

A total of 23 stakeholders were interviewed. Major barriers include the lack of standardized training for cytopathology, the need for new equipment and supplies, and the need to establish a new procedure payment structure for patients. Facilitators included access to global academic partnerships for subspecialty training, availability of cytotechnology training programs, and institutional motivation for change. Participants suggested establishing systematic training in cytopathology, an integrated patient referral pathway for USFNA, appointing an intervention champion, strengthening the cytopathology infrastructure and quality, and modifying payment structures.

**CONCLUSION:**

Multidisciplinary stakeholder involvement, development of rigorous training programs, capacity strengthening of cytopathology infrastructure, and ability to bill for procedures will be critical for the successful adoption of USFNA and ROSE.

## BACKGROUND

Fine-needle aspiration (FNA) is often the primary tissue sampling method for masses in resource-constrained settings, where core needle biopsies are often unavailable or prohibitively expensive.^1,2^ FNA has been shown to be a safe, minimally invasive, and cost-effective procedure with high diagnostic accuracy and low rates of complications when performed by skilled operators.^4–7^ However, in Tanzania, there is high variability in the quality of FNA services across both individual pathologists and different hospitals. The variability stems from multiple issues, including the lack of highly trained personnel, the absence of subspecialty training programs in cytopathology, inadequate laboratory infrastructure, and insufficient quality assurance programs that correlate cytologic diagnoses with follow-up histologic specimens.^8-11^

**CONTEXT**

**Key Objective**
Ultrasound-guided fine-needle aspiration (USFNA) and rapid onsite evaluation (ROSE) are two evidence-based practices that have been shown to improve the quality of cytopathology specimens. We evaluated the barriers and facilitators for implementing the USFNA and ROSE at a national referral hospital in a low-resource setting.
**Knowledge Generated**
Most respondents recognized that fine-needle aspiration (FNA) could be an important tool for the diagnosis of masses. However, the participants reported several challenges in using FNA, including high nondiagnostic rates. Respondents showed a strong interest in improving FNA by adding interventions, such as ROSE and USFNA. However, the lack of specialty training and standardized reporting, the need to procure equipment and supplies, and the need to create financial reimbursement are common barriers for interventional cytology.
**Relevance**
FNA is an important tissue sampling method, particularly in resource-limited settings. The addition of US and/or ROSE has the potential to improve the performance of FNA.

Ultrasound (US) guidance and rapid onsite evaluation (ROSE) are two evidence-based interventions that have been shown to improve the performance of FNA.^12,13^ ROSE is the immediate assessment of a cytologic sample for diagnostic adequacy and can also guide the collection of ancillary tests, such as culture and flow cytometry.^14,15^ In some cases, a preliminary diagnosis can be made and the patient can be immediately referred for additional specialized care.^1^ The addition of US allows real-time visualization of the target lesion and needle path to optimize sampling and diagnosis.^16^ The combination of US and ROSE has the potential to increase overall diagnostic accuracy by up to 97%^17–19,21^ and reduce the number of inadequate samples.^22^ Currently, FNA in Tanzania is primarily performed by palpation and without ROSE, resulting in a high rate of indeterminate or nondiagnostic samples. The introduction of USFNA and ROSE has the potential to improve FNA performance and reduce the number of repeat FNAs and surgical biopsies performed solely for diagnosis and may also reduce costs and delays in cancer diagnosis.

Therefore, we aimed to evaluate the contextual factors affecting the implementation of the USFNA and ROSE in a resource-limited setting. Findings from this formative assessment will help to inform the development of an intervention to introduce the USFNA and ROSE at Muhimbili National Hospital (MNH), the largest referral hospital in Tanzania. Although the implementation of USFNA and ROSE will not resolve all the limitations to pathology services in Tanzania, they will be critical components embedded within broader capacity-strengthening efforts that target multiples aspects of the laboratory.

## METHODS

### Study Design, Setting, and Period

A descriptive qualitative study was conducted to examine gaps in FNA diagnosis, along with barriers and facilitators to implementing USFNA and ROSE at the MNH from 2024 to 2025. Participants were prompted to describe the current challenges with FNA services and to explore multilevel factors influencing the successful implementation of USFNA and ROSE.

The MNH is a national-level referral hospital with a wide range of specialists providing highly specialized services in Tanzania and is the main teaching hospital affiliated with the Muhimbili University of Health and Allied Sciences (MUHAS). MNH receives the highest volume of pathology cases in Tanzania and has a well-established FNA clinic within the Central Pathology Laboratory, where approximately 50 FNA are performed weekly from various anatomic sites. MNH also has a well-established interventional radiology service in the Department of Radiology and Imaging, where approximately 12 core biopsies per week are performed using either US or computed tomography guidance, as well as a dedicated breast radiology clinic, where approximately four US-guided breast core biopsies are performed each week.

### Participants and Recruitment Procedures

Participants were recruited using purposeful sampling to ensure that all key stakeholders involved in FNA services at the MNH were represented (ie, pathologists, surgeons, radiologists, and laboratory scientists). A total of 36 individuals were invited via telephone and email to participate in the study. Eligible participants included key stakeholders with either more than 12 months of employment at the MNH or MUHAS or who were clinical trainees at MUHAS. For trainees, we recruited individuals in their senior rotations (ie, second- or third-year residents or second-year students in the Master of Science in Histotechnology program) or enrolled in a 2-year interventional radiology fellowship program. Participants were required to have had at least one prior experience with either performing or observing an FNA procedure or referring a patient for FNA in their clinical practice. Some study participants also had active administrative roles during study period, ranging from Head of Departments to Head of Units. All participants were permanent residents or citizens of Tanzania and were fluent in Swahili and/or English.

### Data Collection Instruments and Methods

Data were collected through in-depth interviews of key informants using a semistructured interview guide that was developed using the Consolidated Framework for Implementation Research (CFIR; Table 1)^23^ (Appendix). The guide was prepared in English, translated into Swahili, and pilot tested before use. The interviews were conducted in person in a private room in the MNH Central Pathology Laboratory and audio-recorded. The duration of the interviews was approximately 60 minutes. The interviews were conducted by the principal investigator (A.H.K.) and the clinical research coordinator (B.K.).

**TABLE 1:** Selected CFIR Domains and Constructs for ROSE and USFNA Project

Domains	Constructs
Innovation domain	Relative advantage Complexity Adaptability Cost
Individual domain	Individual with informal influence on attitude and behaviors Facilitator: Individual with subject matter expertise
Inner setting	Physical and work infrastructure Communication Tension for changes Capabilities Materials and equipment Access to knowledge and information
Outer setting	Partnership and connections Funding from external source
Process	Teaming Assess the needs and planning

Abbreviations: CFIR, Consolidated Framework for Implementation Research; ROSE, rapid onsite evaluation; USFNA, ultrasound-guided fine-needle aspiration.

### Data Analysis

All interviews were audio-recorded, transcribed, translated into English, and then analyzed by at least two study team members. Thematic analysis was conducted using a deductive rapid analysis approach guided by CFIR, using a rapid analysis matrix.^24^ Data collection and analysis were simultaneously performed to ensure data saturation. Members of the study team (A.H.K., B.K., and Z.I.) first analyzed all 23 interviews using summary templates individually. The team then met to discuss the major themes and transferred the consolidated summaries into a single final summary matrix.

## RESULTS

A total of 23 of the 36 invited stakeholders (64%) from different specialties participated in the interviews, including pathologists, surgeons, radiologists, and laboratory scientists. Most participants were male (15/23, 65%), and 47% (11/23) were pathologists or pathology residents. About half of the participants were trainees (47%, 11/23), and 30% (7/23) of the individuals were staff or faculty with 1 to 5 years of experience. There were 4/23 (17%) of participants who also held administrative positions, including two Heads of Departments and two Heads of Units. Participant characteristics are presented in Table 2.

**TABLE 2:** Characteristic of Study Participants

Characteristic of Study Participant	Number Out of Total, N = 23, No. (%)
Sex	
Male	15 (65)
Female	8 (35)
Specialties	
Pathologist	4 (17)
Surgeon	4 (17)
Interventional radiologist	2 (9)
Laboratory scientist	2 (9)
Pathology resident	7 (30)
Surgeon resident	2 (9%)
Intervention radiology fellow	1 (4)
MSc histotechnology student	1 (4)
Years of experience	
Resident, year 2	5 (22)
Resident, year 3	4 (17)
MSc histotechnology, year 2	1 (4)
Intervention radiology fellow, year 1	1 (4)
1-5 years	7 (30)
6-10 years	2 (9)
>10 years	3 (13)
Participants with administrative roles	
Head of department	2 (9)
Head of unit	2 (9)
No administrative role	19 (82)

Abbreviation: MSc Histotechnology, Master of Science in Histotechnology.

### Current FNA Practice and Its Challenges

All participants felt that FNA was an accessible, minimally invasive, and inexpensive diagnostic test with a shorter turnaround time than core or open tissue biopsies. Participants frequently reported the use of FNA for the preliminary screening of lesions before requesting an open tissue biopsy for confirmation. They also described the practical use of FNA for diagnosis, especially for head and neck lesions, and lesions with a high risk of severe bleeding or scar formation. Pathologists have frequently reported that FNA is useful for the diagnosis of benign breast lesions, such as fibroadenomas. Other participants have reported that FNA can be helpful in diagnosing tumor recurrence. Surgeons have reported that FNA could be helpful for tumor staging, particularly for sampling axillary lymph nodes in patients with breast cancer (Table 3).

**TABLE 3:** List of Barriers, Facilitators, Suggestions for Improvement, and Example of Quotes

Theme	Subtheme	Exemplary Quotes
Barriers		
Challenge of the current FNA practice	High rates of nondiagnostic and inconclusive results	Sometimes this test [FNA] is not conclusive or maybe the sample taken is not representative and often they recommend open biopsy (Surgeon 2)
	Lack of standardized training and reporting	We don't have people who are specialized in cytopathology……and we don't use the standard way of reporting than the rest of the world (Pathologist 5)
	Lack of trust for cytology results among clinician	FNA results may not always be the same as the final tissue diagnosis. That is one of the reasons some of the surgeons do not fully trust fine-needle aspiration results (Surgeon 4)
Inner setting/capacity	Lack of equipment and supplies of as well as associated maintenance and calibration	A challenge is to have ultrasound machines which will be available at the fine-needle aspiration clinic all the time just like in the radiology clinic (Pathologist 8) The obvious barriers for ROSE will be availability of the reagents and microscope (Pathologist 8)
	Understaffing	Availability of pathologists for rapid onsite because they are few (Pathologist 10)
	Challenges with instrument care and chain of custody for equipment in the procedure to be done in the wards	Challenges with instrument care and chain of custody (eg, microscope used during FNA) in the wards (Surgeon 1)
Inner setting/infrastructure	Inadequate infrastructure and space for imaging, sampling, and placement of equipment	The clinic space is really limited from my experience in our clinic; the room is small (Pathologist 1) I don't think we have enough equipment to make sure we collect the right way, there are challenges with ventilation, air conditioning, privacy, and shortage of proper syringe, needle, and FNA gun (Pathologist 6) For guided FNA, we are facing challenges because of the competition on ultrasound machine used by radiologist (laboratory scientist 2)
Inner setting/workflow	Increase in procedure time	It is time consuming especially when the flow of patient is huge and you are performing both, fine-needle aspiration under ultrasound guidance and ROSE (Pathologist 3)
	Conflict of interest in referral pathway that necessitates the need to establish new referral system for FNA and core needle procedures	Maybe to the department of radiology, they might see that you are taking their job because they are the ones who do guided procedure (Laboratory scientist 1) If ROSE is going to be performed in pathology and radiology, there will be an interdepartmental competition on allocation of resources (microscope) (Pathology 8)
	Laboratory standard operating procedures are not systematically created or routinely followed	There is need to adhere to the protocol or standards of using ROSE (pathologist 3) Another challenge [is] if there is an introduction of these new technologies there must be a launching of the new SOPs on how to perform ultrasound-guided fine-needle aspiration, so that means we will have to change our SOP (Laboratory scientist 3)
Work flow/culture/	Expectation of incentives to perform extra tasks will cause resistance to change	I think the challenges are willingness of people to adopt and learn a new intervention (Pathologist 5)
Training and expertise	No standardized training for USFNA or ROSE, and lack of faculty with subspecialized training in cytopathology	There is a need to have standardized training, if there isn't enough training in relation to ROSE, it can probably be a challenge (Pathologist 7) Many pathologists have not been trained to operate radiologic tools, especially ultrasound (Laboratory scientist 2)
Financing	Additional cost for USFNA and ROSE procedures, and for their training	If you add rapid onsite test, it's going to add the cost of the whole service, so it might raise the FNA price (Pathologist 1)
	Need to establish a billing and reimbursement mechanism for USFNA and ROSE	When clinicians order a patient for fine-needle aspiration, it should be made clear that it is fine-needle aspiration under ultrasound guidance, so cost of ultrasound guidance should be included (Laboratory scientist 3)
Facilitators		
Perceived positive impact on patient care	Enhance diagnostic ability	ROSE will make sure pathologists have sufficient sample for diagnosis and improve diagnostic precision (Pathologist 1) ROSE will help in making FNA to be a more reliable test (Pathology 8) USFNA will improve diagnostic modalities (Pathology 1)
Perceived positive impact on patient care	Increase FNA accuracy	Ultrasound will help in visualization and spotting area of interest and hence improve accuracy (Pathologist 7)
	Reduction in cost to patient care	ROSE will reduce multiple sampling procedure for patients and by doing so we also save resources (Laboratory scientist 2)
Partnership for development	Access to donor and global academic partnerships for research funding and training	To see if there is access to donor or sponsor who can fund the project once they know the importance, for example, the way Muhimbili Hospital is receiving funding package for research project (Pathologist 8)
Training	Availability of cytotechnology master's program at MUHAS	We should produce more expertise………as cytotechnologists, it should be a continuous and as an official program…………we will see the impact (Radiologist 15)
Tension for change	Existence of long-term tension for change on the quality of pathology reports by clinicians Current demand for improvement of pathology services and desire for pathology laboratory accreditation Current demand to improve cancer care and noncommunicable disease and to establish FNA service in regional referral hospital in Tanzania	Reporting of tissues and cytology depends on pathologist. At other times, we get different results even when you are sure that you have taken a sample from an exact location, when it is read by different pathologists, the results come out different, so those are the challenges (Interventional radiologist 3) If you perform ROSE and you provide good samples and good slides, I think your accreditation will be easily achieved (Pathology 5) I believe that there will be findings that will have a positive impact on the country because we really need cytology especially at the moment cancer and other noncommunicable diseases are increasing (Histotechnologist 3) In the near future, we will need fine-needle or core needle biopsy at health centers not only in national hospitals…because many of our patients because of their income they don't reach to national referral hospital (Histotechnologist 3)
Suggestions for improvement		
Capacity	Strengthen pathology capacity by improving FNA clinic space, availability of equipment, and quality management system needed for USFNA and ROSE procedures	We need to have a well-organized fine-needle aspiration clinic or a laboratory that will have enough space and all the required infrastructure there (Pathologist 9) We need to have the tools; the machines including the ultrasound itself but also the microscope, the reagents, and the rest of the items (pathologist 9)
Training	Provide systematic training in cytopathology, USFNA and ROSE procedures, and cytology interpretation	We need to have sustainable kind of workshop or training to the personnel including the pathologists and also the radiologists so that we can improve the performance (Pathologist 9)
Curriculum	Curriculum review, modification, and addition of course focused on cytology to enhance the quality of cytology training and services in Tanzania	Most procedures are done by the residents who are not permanent members of the hospital, so even if you training a batch of residents in two to three years you need to do more training for each coming batch, probably you might need to think about changing the curriculum (Surgeon 1) To start teaching and making cytopathologists and cytopathology course (Pathology 3)
Pilot	Pilot test USFNA and ROSE gradually and tailor the interventions depending on the need of specific clinic	I think you should be focused on one organ and seeing how it goes, if you see positive results, then you can add other organs (Interventional radiologist 17)
Other suggestions	Establish an integrated patient referral, communication, and follow-up pathways across cancer care services Create and encourage participants to follow SOPs designed for ROSE and USFNA procedures Modify payment scheme for the diagnostic procedure (ie, a bundled package as opposed to individual components) Advocacy and endorsement of USFNA and ROSE by the department, through interdepartmental collaboration and the hospital management Need of feedback mechanism between clinicians Need for champions and implementors	Proper communication among clinician and patient needs to be educated by clinician with regard to the sample results (Surgeon 5) We need a SOP where every person who is doing the procedure will help to follow that SOP, because without a SOP, most of the things will be forgotten (Pathology 10) We need to increase payment for this service so that to create financial safety for the development of this service (Pathology 8) Multidisciplinary approach like involve other departments so as they can be able to support the intervention (Pathology 3) Collaboration between pathologist and surgeon during biopsy taking to provide preliminary diagnosis (Surgeon 3) Provision of feedback to radiologists after a procedure. Not only negative feedback but also positive feedback (Intervention radiology 1) To have dedicated implementors or champion (Intervention radiologist 3)

Abbreviations: FNA, fine-needle aspiration; MUHAS, Muhimbili University of Health and Allied Sciences; ROSE, rapid onsite evaluation; SOP, standard operating procedure; US, ultrasound; USFNA, ultrasound-guided fine-needle aspiration.

The reported challenges in using FNA include higher rates of nondiagnostic or inconclusive results than open biopsies. Other challenges reported by pathologists include an inability to obtain optimal syringes and needles for FNA and a small clinical space for procedures. Pathologists also stated that the lack of specialized and standardized training in FNA and the lack of adherence to international reporting systems are issues that affect the quality of FNA services. Surgeons also frequently reported that they did not trust the accuracy of the cytology reports (Table 3).

### Barriers to Implementing USFNA and ROSE

#### Laboratory Capacity and Infrastructure

Inadequate infrastructure was frequently mentioned as a barrier to implementing ROSE and USFNA. All participants reported a current lack of supplies and equipment for ROSE. In particular, the department has an insufficient number of well-maintained microscopes and some pathologists felt that they could not spare a microscope for ROSE. Common stains for ROSE, such as Diff-Quik and toluidine blue, are difficult to obtain. An additional barrier to implementing USFNA includes the need to purchase, maintain, and store ultrasound equipment in the pathology department. Other barriers to both ROSE and USFNA include the anticipated increase in workload for pathology and radiology staff for an already overstretched and understaffed health care workforce. Many pathologists have also reported the need for cytopathology subspecialty training, including dedicated and systematic training in the ROSE and USFNA. One resident in the surgery ward was unsure of the workflow for FNAs that might be performed in the surgical wards. In particular, it was unclear whether there was a microscope on the surgical wards for ROSE and who would be responsible for maintaining it (Table 3).

#### Laboratory and Clinic Workflow

Increased procedure time was the most frequent concern reported by pathologists and radiologists. Other reported barriers included current suboptimal laboratory practices, including variable adherence to standardized operating procedures, variable use of cytology reporting systems, and nonroutine correlation with radiologic findings. Another major barrier involves interdepartmental politics. In particular, because both the clinical division of interventional radiology and its fellowship were only recently established at MUHAS and MNH, some participants mentioned that radiologists would not likely support the USFNA service provided by the pathology department. Moreover, participants reported an overall lack of motivation and willingness to change among many staff and faculty members, and emphasized the need to establish incentives to implement new practices (Table 3).

#### Innovation Cost and Financial Structuring

Pathologists and radiologists frequently expressed concerns regarding the additional costs associated with performing ROSE and USFNA. They also felt that increased training requirements for these two techniques could hinder their implementation. In addition, participants emphasized the need to establish a clear payment structure for the procedures, pointing out the current lack of sustainable funding (Table 3).

### Facilitators to Implementing USFNA and ROSE

#### Perceived Positive Impact of USFNA and ROSE on Patient Care

Most participants felt that ROSE and USFNA could potentially affect patient care by improving the quality of FNA sampling, reducing the number of repeat and false-negative FNA results, and reducing diagnostic costs. Pathologists felt that ROSE and USFNA would increase their precision and reporting, which, in turn, could improve patient management (Table 3).

#### Partnership for Funding and Training

Participants identified the presence of multidisciplinary global academic partnerships that were available as resources to support the identification of funding sources and the implementation of subspecialty training in cytopathology. Other facilitators include the existence of a multidisciplinary institutional partnership between surgeons, oncologists, pathologists, and radiologists for comprehensive cancer treatment, which facilitates better communication between clinical specialties. A Master of Science in Cytotechnology was also recently established at MUHAS and may be able to facilitate the training of cytotechnologists who may be able to take on some of the tasks related to USFNA and ROSE (Table 3).

#### Institutional Tension for Change

Although some participants reported an overall culture of resistance to change, others highlighted the emerging tension for change. Examples include the establishment of new multidisciplinary working groups and subspecialty tumor boards. Ongoing efforts to achieve accreditation for the anatomic pathology laboratory at the MNH and MUHAS have also been cited. Laboratory scientists mentioned that nationally, there was a high demand for FNA services in regional referral hospitals, where histology is not available. Moreover, the Ministry of Health has identified cancer care as a priority that may support initiatives to strengthen pathology services (Table 3).

#### Suggestions for Successful Implementation of USFNA and ROSE

Participants felt that it was important to ensure the availability of US equipment for USFNA and microscopes as well as reagents for ROSE. In addition, pathologists have recommended dedicated and systematic training for USFNA, ROSE, and cytology interpretation. Pathologists have also suggested that to improve cytopathology services, the overall quality management system needs to be strengthened, including creating and adhering to standard operating procedures and systematically conducting cytologic-histologic correlations. Radiologists suggested piloting the USFNA and ROSE in a stepwise fashion, for example, selecting a specific organ system, such as the thyroid, and tailoring interventions based on the needs of the specific clinic. Several participants suggested establishing better patient referral pathways and improving communication among clinical subspecialties. Other suggestions included receiving approval and endorsement by hospital and departmental management and collaborating with other departments to implement the interventions. Pathologists also suggested modifying the current payment structure for diagnostic procedures, that is, a bundled package as opposed to individual components, to make diagnostic costs more apparent upfront for patients. To support sustainability, a specific budget or external funds may be required. Incentives to motivate pathologists and radiologists to adopt interventions and make changes may also be required (Table 3).

## DISCUSSION

In this study, we elucidated the contextual factors that influence the implementation of the USFNA and ROSE in the MNH. Our findings note a strong interest in introducing USFNA and ROSE among stakeholders across clinical specialties. However, we identified several major barriers to implementing ROSE and USFNA. These included a preference by clinicians for core needle and open tissue biopsies over FNA, inadequate equipment, difficulty in obtaining commonly used rapid stains, a lack of subspecialty training programs in cytopathology, the impact on clinical workflows and procedure costs, and the need to modify the payment and reimbursement structure for FNAs. Prior publications noted similar barriers in equipment, supplies, training, workflows, and cost of implementing improvements to pathology services, including USFNA, in a range of resource settings.^25-33^ Facilitators identified in our study included access to global academic partnerships for training and support in identifying external funding sources, the existence of multidisciplinary comprehensive cancer care groups at the MNH to facilitate communication and collaboration, access to a training program for cytotechnologists, and a general tension for change among stakeholders. A previous study noted similar facilitators, including the presence of strong support systems, good collaboration and working relationships between pathology and radiology, the availability of formal training in cytopathology, easy-to-use US, routine maintenance of equipment, and strong motivations to change among stakeholders.^26-28^

By identifying barriers, our group can consider potential solutions early in intervention. For example, instead of using more expensive, larger US units that are difficult to store, portable tablet-based US devices may be used. Procuring portable microscopes that are sufficient for ROSE may also be an alternative to bulky light microscopes used for diagnosis. Although common rapid stains such as toluidine blue and Diff-Quik can be difficult to source in Tanzania, modified protocols of available stains, such as rapid hematoxylin and eosin, can be explored.^34^ Training key stakeholders on understanding when USFNA and ROSE are appropriate, as well as pathologists in advanced sampling techniques, morphologic interpretation, and the need for radiologic correlation, will be fundamental for successful implementation.^35,36^ Of note, intensive workshops will be helpful in transferring basic skills in USFNA, but additional hands-on and supervised training with frequent feedback are required for mastery.^37^ Strengthening the existing laboratory quality management system will also help standardize all cytopathologic processes, including specimen reporting. Although pathologists were concerned about the increased amount of time ROSE would add to the procedure, cytotechnologists may help expedite the procedures and may even perform ROSE.^38-40^ Moreover, reducing the number of repeated procedures also reduces the workload in the long term. The participants were also concerned about the need to establish new referral pathways for patients. However, the existence of multidisciplinary comprehensive cancer care groups will help establish protocols and streamline the identification of patients for USFNA. Billing and modification of the payment structure to integrate USFNA and ROSE are also critical strategies for scaling up and sustainability.^12,27,41^ Although the individual cost for each procedure may increase if US and ROSE are used, we anticipate that by reducing the need for repeat or more invasive diagnostic procedures, the overall average costs should be reduced. However, a dedicated cost-impact study needs to be conducted.

This study has several limitations. First, purposeful sampling inherently has selection bias. Although we attempted to enroll health care workers from a range of specialties, most participants were pathologists or pathology trainees. Moreover, there were also a limited number of high-level hospital administrators enrolled; however, several Heads of Departments and Heads of Unit were included in the current cohort. Participants who were enrolled in the study were also more likely to be available and interested in new interventions. Despite these limitations, this study was strengthened by sampling from diverse disciplines, backgrounds, and experiences, to provide the most representative data. To increase research rigor, we used a semistructured interview guide, and all interviews were conducted by the principal investigator, who was well trained in qualitative research. The data were analyzed using a thematic approach by the reach team to maintain consistency. The results may also not be generalizable to other hospitals in East Africa, since the MNH is a tertiary care hospital affiliated with a university and has the largest public pathology laboratory in the country.

In conclusion, this study identified the eagerness to improve cytopathology services in Tanzania. Multidisciplinary stakeholder engagement is required to develop an effective and sustainable implementation strategy for these two evidence-based practices. Future studies focusing on implementation, clinical outcomes, and financial impact will further advance our understanding of how to improve and disseminate evidence-based practices.

## APPENDIX

### Semistructured Interview Guide for Stakeholder

Inner setting and individual characteristic
  1. Tell me about fine-needle aspiration biopsy services at your institution. (Probe: In what scenarios are patients referred for FNAB? What is the FNAB referral process? Who performs the FNAB?)
  2. What are your thoughts on FNAB for tumor diagnosis? (Probe: Fear of false-positive results? Use of FNAB for stratifying patient who were not fit for open tissue biopsy for further treatment decision? Fear of inconclusive results?)
  3. Tell me about core needle biopsy services at your institution. (Probe: In what scenarios are patients referred for FNAB? What is the CNB referral process? Who performs the CNB?)
  4. What are your thoughts about core needle biopsy for tumor diagnosis? (Probe: Fear of false-positive results? Insufficient core due to tumor necrosis, satisfaction of core reports?)
Inner setting
  5. In what situations do patients skip FNAB and go directly for core biopsy or surgical excision for diagnosis?
  6. How would you change the current FNAB services at your institution?
Process
  7. We are conducting a study on how to best improve FNAB and cytology services for cancer diagnosis. Two interventions we are considering are rapid onsite evaluation (ROSE) and ultrasound (US). Tell me what you know about ROSE.
  8. How do you think ROSE would change FNAB procedures compared with the current practice at your institution? (Probe: What impact on diagnosis or clinical care will it have?)
  9. What are the barriers for using ROSE in FNAB?
  10. What are the barriers for using ROSE in core needle biopsy?
    Probes: Qs9&10
    - Lack of skills, knowledge, or awareness
    - Intensive training needed
    - Time-consuming for laboratory staff
    - Increased wait time for patients
    - Equipment and reagents
    - Cost
    - Insufficient motivation among personnel
    - Unable to integrate into current clinical or laboratory workflows
  11. Tell me about ultrasound-guided biopsy procedures at your institution.
  12. How do you think having US in the cytology clinic would change FNAB procedures compared with the current practice at your institution? (Probe: What impact on diagnosis or clinical care will it have?)
  13. What are the barriers to using US in an FNAB clinic or US in core needle biopsy sampling?
    Probes:
    - Lack of skills, knowledge, or awareness
    - Intensive training needed
    - Time-consuming for staff
    - Increased waiting time to patients
    - Lack of local repair options
    - Cost
    - Insufficient motivation among personnel
    - Unable to integrate into current clinical or laboratory workflows
  14. What are the barriers for using both ROSE and US jointly during either FNAB or CNB?
  15. What are some ways that these barriers could be overcome so that these interventions can be sustainable in the long term?
Outer setting
  16. What policies and regulations are needed to adopt a new intervention in your institution?

#### Final wrap-up questions

Finally, do you have any other suggestions or recommendation with regard to the use of ROSE and ultrasound (US) to improve FNAB services?

Remarks: The discussion was so very helpful. Thank you for your time. You may be contacted for follow-up interviews.

## References

[1] Schmidt RL Witt BL Lopez-Calderon LE et al The influence of rapid onsite evaluation on the adequacy rate of fine-needle aspiration cytology: A systematic review and meta-analysis Am J Clin Pathol 139 300 308 2013 23429365 10.1309/AJCPEGZMJKC42VUP

[2] Kabadi JL Correlation Between Fine Needle Aspiration Cytology (FNAC) and Surgical Biopsy in Tissue Diagnosis of Breast Lumps at Muhimbili National Hospital, Dar es Salaam, Tanzania Thesis University of Dar es Salaam 2002 http://41.86.178.5:8080/xmlui/handle/123456789/6062

[3] Reference deleted

[4] Silva WPP Stramandinoli-Zanicotti RT Schussel JL et al Accuracy, sensitivity and specificity of fine needle aspiration biopsy for salivary gland tumors: A retrospective study from 2006 to 2011 Asian Pac J Cancer Prev 17 4973 4976 2016 28032725 10.22034/APJCP.2016.17.11.4973 PMC5454705

[5] Pinchot SN Al-Wagih H Schaefer S et al Accuracy of fine-needle aspiration biopsy for predicting neoplasm or carcinoma in thyroid nodules 4 cm or larger Arch Surg 144 649 655 2009 19620545 10.1001/archsurg.2009.116 PMC2910711

[6] Yu YH Wei W Liu JL Diagnostic value of fine-needle aspiration biopsy for breast mass: A systematic review and meta-analysis BMC Cancer 12 41 2012 22277164 10.1186/1471-2407-12-41 PMC3283452

[7] Sinna EA Ezzat N Diagnostic accuracy of fine needle aspiration cytology in thyroid lesions J Egypt Natl Cancer Inst 24 63 70 2012 10.1016/j.jnci.2012.01.001 23582597

[8] Sayed S Cherniak W Lawler M et al Improving pathology and laboratory medicine in low-income and middle-income countries: Roadmap to solutions Lancet 391 1939 1952 2018 29550027 10.1016/S0140-6736(18)30459-8

[9] Frech S Bravo LE Rodriguez I et al Strengthening pathology capacity to deliver quality cancer care in cities in LMICs JCO Glob Oncol 10.1200/GO.20.00604 10.1200/GO.20.00604 PMC8457877 34129368

[10] Folaranmi OO Olayiwola OI Ibiyeye KM et al Cytopathology practice in Nigeria Diagn Cytopathol 53 186 190 2025 39739999 10.1002/dc.25441

[11] Yadav K Cree I Field A et al Importance of cytopathologic diagnosis in early cancer diagnosis in resource-constrained countries JCO Glob Oncol 10.1200/GO.21.00337 10.1200/GO.21.00337 PMC8887942 35213215

[12] Michael CW Kameyama K Kitagawa W et al Rapid on-site evaluation (ROSE) for fine needle aspiration of thyroid: Benefits, challenges and innovative solutions Gland Surg 9 1708 1715 2020 33224848 10.21037/gs-2019-catp-23 PMC7667097

[13] Schmidt RL Witt BL Matynia AP et al Rapid on-site evaluation increases endoscopic ultrasound-guided fine-needle aspiration adequacy for pancreatic lesions Dig Dis Sci 58 872 882 2013 23053888 10.1007/s10620-012-2411-1

[14] Philipo GS Vuhahula E Kimambo A et al Feasibility of fine-needle aspiration biopsy and rapid on-site evaluation for immediate triage in breast cancer screening in Tanzania JCO Glob Oncol 10.1200/GO.20.00279 10.1200/GO.20.00279 PMC8081537 33493018

[15] Huang Z Zhuang D Feng A et al Real-time and accuracy of rapid on-site cytological evaluation of lung cancer Transl Cancer Res 10 479 486 2021 35116277 10.21037/tcr-20-3294 PMC8798460

[16] for the European Society of Breast Imaging (EUSOBI), with language review by Europa Donna–The European Breast Cancer Coalition Bick U Trimboli RM et al Image-guided breast biopsy and localisation: Recommendations for information to women and referring physicians by the European Society of Breast Imaging Insights Imaging 11 12 2020 32025985 10.1186/s13244-019-0803-x PMC7002629

[17] Halenka M Fryšák Z Ultrasound-guided fine-needle aspiration biopsy (US-FNAB) Halenka M Fryšák Z Atlas of Thyroid Ultrasonography Berlin Springer International Publishing 2017 383 392

[18] Danese D Sciacchitano S Farsetti A et al Diagnostic accuracy of conventional versus sonography-guided fine-needle aspiration biopsy of thyroid nodules Thyroid 8 15 21 1998 9492148 10.1089/thy.1998.8.15

[19] Singh Ospina N Brito JP Maraka S Diagnostic accuracy of ultrasound-guided fine needle aspiration biopsy for thyroid malignancy: Systematic review and meta-analysis Endocrine 53 651-661 2016 27071659 10.1007/s12020-016-0921-x

[20] Reference deleted

[21] Brancato B Crocetti E Bianchi S et al Accuracy of needle biopsy of breast lesions visible on ultrasound: Audit of fine needle versus core needle biopsy in 3233 consecutive samplings with ascertained outcomes Breast 21 449 454 2012 22088803 10.1016/j.breast.2011.10.008

[22] Qi JC Liao L Zhao Z et al Impact of rapid on-site evaluation combined with endobronchial ultrasound and virtual bronchoscopic navigation in diagnosing peripheral lung lesions BMC Pulm Med 22 117 2022 35361163 10.1186/s12890-022-01917-z PMC8969361

[23] Means AR Kemp CG Gwayi-Chore MC et al Evaluating and optimizing the consolidated framework for implementation research (CFIR) for use in low- and middle-income countries: a systematic review Implement Sci 15 17 2020 32164692 10.1186/s13012-020-0977-0 PMC7069199

[24] Nevedal AL Reardon CM Opra Widerquist MA et al Rapid versus traditional qualitative analysis using the Consolidated Framework for Implementation Research (CFIR) Implement Sci 16 67 2021 34215286 10.1186/s13012-021-01111-5 PMC8252308

[25] Rabin TL Mayanja-Kizza H Rastegar A Medical education capacity-building partnerships for health care systems development AMA J Ethics 18 710 717 2016 27437821 10.1001/journalofethics.2016.18.7.medu2-1607

[26] Lynn TJ Monaco SE Adding the “US” in FNA: Pearls for starting a pathologist-performed ultrasound-guided fine needle aspiration service (USG FNA) Semin Diagn Pathol 39 410 420 2022 35718580 10.1053/j.semdp.2022.06.007

[27] Ljung BM Pitman MB Establishing ultrasound-guided fine-needle aspiration in an academic setting Pathol Case Rev 18 48 52 2013

[28] Suttels V Guedes Da Costa S Garcia E et al Barriers and facilitators to implementation of point-of-care lung ultrasonography in a tertiary centre in Benin: A qualitative study among general physicians and pneumologists BMJ Open 13 e070765 2023 10.1136/bmjopen-2022-070765 PMC10357755 37369423

[29] Lynch KA Omisore AD Famurewa O et al Designing participatory needs assessments to support global health interventions in time-limited settings: A case study from Nigeria Int J Qual Methods 20 16094069211002421 2021 10.1177/16094069211002421 PMC8855957 35185442

[30] Andiric LR Massambu CG Laboratory quality improvement in Tanzania Am J Clin Pathol 143 566 572 2015 25780009 10.1309/AJCPAB4A6WWPYIEN

[31] Andiric LR Massambu CG One laboratory’s progress toward accreditation in Tanzania Afr J Lab Med 5 1 4 2016 10.4102/ajlm.v3i2.202 PMC5637807 29043185

[32] Schneidman M Matu M Nkengasong J et al Building cross-country networks for laboratory capacity and improvement Clin Lab Med 38 119 130 2018 29412876 10.1016/j.cll.2017.10.009

[33] Sayed S Cherniak W Lawler M et al Pathology and laboratory medicine in low-income and middle-income countries 2: Improving pathology and laboratory medicine in low-income and middle-income countries: Roadmap to solutions Lancet 391 1939 1952 2018 29550027 10.1016/S0140-6736(18)30459-8

[34] Agarwal P Toi PC Subramaniam H et al Prospective comparison of cytological specimen adequacy assessment by different rapid staining techniques for rapid on‐site evaluation in fine needle aspiration cytology and their cost‐effectiveness Diagn Cytopathol 47 469 474 2019 30585436 10.1002/dc.24139

[35] Lynch KA Omisore AD Atkinson TM et al Multistakeholder needs assessment to inform the development of an mHealth-based ultrasound-guided breast biopsy training program in Nigeria JCO Glob Oncol 10.1200/GO.20.00353 10.1200/GO.20.00353 PMC7713594 33216646

[36] Ng DL Vuhahula E Zhang L et al Efficacy of an intensive, ultrasound-guided fine-needle aspiration biopsy training workshop in Tanzania J Glob Oncol 4 1 9 2018 10.1200/JGO.18.00134 PMC7010453 30372401

[37] Yang SR Ljung BM Teaching and learning FNA biopsy: An update for the modern audience Cancer Cytopathol 127 615 617 2019 31433563 10.1002/cncy.22169

[38] Selvaggi SM On the job training: An educational program in ROSE of fine needle aspirates and telecytology for cytotechnologists J Am Soc Cytopathol 7 306 310 2018 31043300 10.1016/j.jasc.2018.05.004

[39] Tinnirello AA Rapid on-site evaluation of fine-needle aspiration specimens using cytotechnologist-performed telecytology: Insights and advantages Cytopathology 36 2 11 2025 39158137 10.1111/cyt.13428

[40] Collins JA Novak A Ali SZ et al Cytotechnologists and on-site evaluation of adequacy Korean J Pathol 47 405 410 2013 24255627 10.4132/KoreanJPathol.2013.47.5.405 PMC3830986

[41] Wu M Shroyer KR Strategies for building a successful ultrasound guided FNA practice in department of pathology—Experience at a university hospital Diagn Cytopathol 45 878 882 2017 28699306 10.1002/dc.23784

